# The vascular mimic: successful surgical management of cavernous sinus hemangioma via adapted approach: Case report

**DOI:** 10.1097/MD.0000000000045254

**Published:** 2025-10-17

**Authors:** Mohanad A. Abuzahra, Malak Hroub, Mohammed A. Barakat, Omar H. Salloum, Omar Hamadi, Maha Ramzi

**Affiliations:** a Department of Radiology, Istishari Arab Hospital, Ramallah, Palestine; b Faculty of Medicine, Al-Quds University, Jerusalem, Palestine; c Department of Radiology, Rafidia Hospital, Nablus, Palestine; d Faculty of Medicine, Palestine Polytechnic University, Hebron, Palestine; e Department of Internal Medicine, Advocate Illinois Masonic Medical Center, Chicago, IL; f Faculty of Health Professions, Al-Quds University, Jerusalem, Palestine.

**Keywords:** case report, cavernous sinus hemangioma, craniotomy, subtotal resection

## Abstract

**Background:**

Cavernous sinus hemangioma (CSH) is a rare, benign vascular tumor presenting significant diagnostic and management challenges due to its location and mimicry of other sellar and parasellar masses. This report details such a case, emphasizing the diagnostic complexities and surgical adaptability required.

**Patient concerns:**

A 47-year-old female presented with 3 months history of progressive left eye visual decline, intermittent headaches, and neck pain radiating to the left upper limb.

**Diagnoses:**

Imaging revealed a large sellar and parasellar mass with mass effect upon adjacent structures.

**Interventions:**

Surgical management was initially via an endoscopic transnasal transsphenoidal approach; however, intraoperative findings of high vascularity and cerebrospinal fluid leakage necessitated conversion to a left pterional craniotomy with subtotal resection of this mass. Histopathology confirmed the diagnosis.

**Outcomes:**

Postoperatively, the patient had a good general outcome and remains under regular scheduled follow-up.

**Lessons:**

Despite being rare, CSH should be considered among the causes of sellar and parasellar masses. Its location can lead to misdiagnosis, but imaging studies and a multidisciplinary team approach can decrease the risk of complications in such cases. While CSH are considered benign vascular tumors, they are associated with the risk of significant blood loss with surgery. This case highlights the importance of preoperative planning, intraoperative adaptability, and a multidisciplinary approach in managing large vascular brain tumors. Early diagnosis and appropriate surgical intervention can significantly improve clinical outcomes in complex cases.

## 1. Introduction

Sellar and parasellar tumors present considerable diagnostic and surgical challenges due to their proximity to critical neurovascular structures, including the optic nerves, hypothalamus, pituitary gland, internal carotid artery (ICA), and cavernous sinuses.^[1]^ While pituitary adenomas and meningiomas are considered the most common lesions in this region, other rare pathologies, such as cavernous sinus hemangioma (CSH)s – benign vascular malformations – require tailored management approaches associated with high vascularity, posing additional surgical risks.^[1]^ Accurate preoperative diagnosis, as noted in many cases in the literature,^[2,3]^ is crucial but challenging.

These diagnostic and management challenges are often compounded in underserved regions, where access to advanced imaging and multidisciplinary teams may be limited.^[4]^

We present the case of a 47-year-old female with a large, highly vascular sellar and parasellar tumor initially suspected to be a different entity, highlighting the diagnostic and surgical hurdles encountered.

## 2. Methods

This work has been reported in accordance with CARE guidelines.

Ethical Approval: All aspects of the study protocol, including access to and use of the patient’s clinical information, were authorized by our institution which is responsible for the patient. Our institution does not require ethical approval specifically for case reports that involve routine clinical care and standard interventions.

Consent: Written informed consent was obtained from the patient for the publication of this case report and accompanying images. A copy of the written consent is available for review by the Editor-in-Chief of this journal on request.

### 2.1. Case presentation

A 47-year-old woman presented to the outpatient clinic with complaints of newly occurring intermittent headaches over the past few weeks, described as dull and localized to the frontal region, without associated nausea, vomiting, or photophobia. She also reported a progressive decline in vision in her left eye, with a current visual acuity of 6/24. She denied any episodes of diplopia, eye pain, or redness.

There were no associated symptoms of seizures, focal neurological deficits, altered consciousness, or behavioral changes. She denied any weakness, numbness, gait disturbances, or sphincter incontinence.

Her past medical history was unremarkable, with no history of hypertension, diabetes, or autoimmune diseases. She was not on any regular medications. There was no personal or family history of malignancies, nor any known genetic disorders.

### 2.2. Diagnostic assessment

On physical examination, the patient was fully conscious and oriented, neurological examination showed left eye ptosis and pupil dilation, indicative of oculomotor palsy. Blurring of vision was detected in the left eye, with finger counting possible at a 2-m distance. Ophthalmoscopy revealed mild optic nerve pallor. Right eye vision remained functional (6/6). There were no other focal neurological deficits. Hormonal profiles, including cortisol, TSH, and prolactin, were within normal ranges.

**Figure 1.**
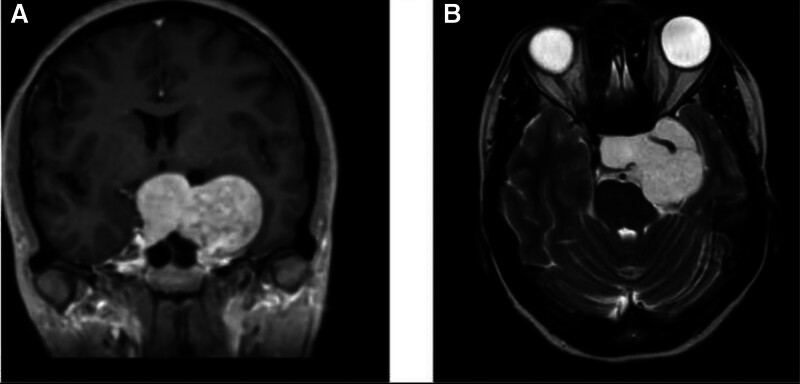
MRI scan of the mass including coronal T1 post-contrast (A) and axial T2-weighted images (B) demonstrating a well-defined extra-axial sellar and left parasellar mass with avid homogenous enhancement encircling the left internal carotid artery. MRI = magnetic resonance imaging.

**Figure 2.**
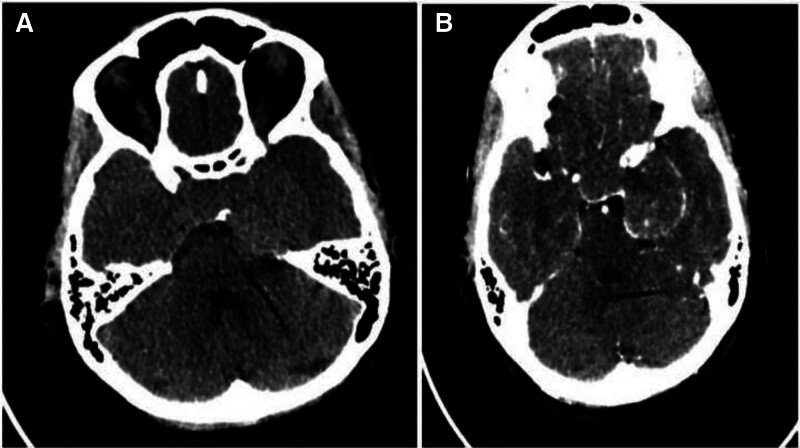
CT scan of the mass including non-contrasted (A) and contrasted images (B), these showed isodense mass centered in the left cavernous sinus with no bony destruction. Arterially enhancing components are noted within the mass. CT = computed tomography.

Brain CT and MRI revealed a large, extra-axial sellar and left parasellar vascular mass (5.3 × 4.2 × 3.6 cm), compressing the left optic nerve and encircling the cavernous portion of the left ICA. On MRI, the mass exhibited high signal intensity on T2-weighted images and demonstrated characteristic progressive “fill-in” contrast enhancement with intense homogeneous enhancement on late gadolinium-enhanced T1-weighted images. These features were pivotal in suspecting a CSH. Notably, despite encasing the left ICA, there was no evidence of luminal narrowing, a feature often seen in meningiomas. The remaining dural venous sinuses were patent with no evidence of thrombosis. No hydrocephalus, midline shift, or intracranial hemorrhage was noted. Bone structures appeared intact (Figs. 1 and 2).

### 2.3. Therapeutic intervention

**Figure 3.**
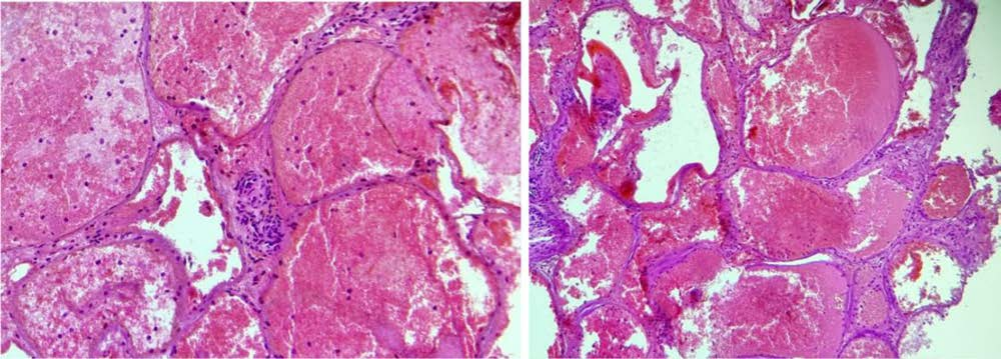
Histopathology slice showing a benign vascular lesion consisting of blood-filled cavernous spaces, consistent with a cavernous hemangioma of the sellar and parasellar region, with no evidence of malignancy.

An endoscopic transnasal transsphenoidal approach was initially chosen due to its minimally invasive nature and direct access for sellar lesions, aiming primarily to decompress the optic apparatus through debulking the sellar mass; a major factor of the patient vision deterioration, despite this initial choice and acknowledging its potential inadequacy for large or parasellar-extending lesions, the surgical team was prepared for alterations once the intraoperative findings revealed a highly vascular tumor leading to CSF leakage and significant bleeding, which necessitated a change in approach. Consequently, the approach was converted to a left pterional craniotomy for tumor biopsy and partial resection. Despite this initial choice and acknowledging its potential inadequacy for large or parasellar-extending lesions, intraoperative findings revealed a highly vascular tumor leading to CSF leakage and significant bleeding, which necessitated a change in approach. Consequently, the approach was converted to a left pterional craniotomy for tumor biopsy and partial resection. Histopathological examination confirmed a benign cavernous hemangioma (Fig. 3).

### 2.4. Follow-up and outcomes

Postoperatively, the patient was hemodynamically stable, promptly extubated, and transferred to the ICU for standard neurosurgical monitoring. A postoperative CT scan confirmed expected surgical changes with a mild midline shift and no acute complications.

Showing progressive neurological recovery and stable vitals, the patient was subsequently moved from the ICU to the neurosurgical ward. Once discharge criteria were met, the patient was sent home with arrangements for outpatient neurosurgical follow-up.

The patient showed a favorable short-term outcome, marked by successful surgery and steady progression. Although residual nerve symptoms persisted – including left eye ptosis and a dilated pupil – these gradually improved over the following 9 months of follow-up, sustaining good neurological recovery.

## 3. Discussion

Cavernous sinuses are paired extra-axial venous plexuses located in the middle cranial fossa and lined by dura mater; they contain various venous channels and are closely associated with the ICA, several cranial nerves, and the sympathetic plexus.^[1,5]^ Tumors in these sinuses are likely to present with overlapping clinical symptoms, emphasizing the necessity of imaging for accurate diagnosis, extent assessment, differential diagnosis, and biopsy planning.^[6]^

CSHs are slow-growing benign vascular malformations that cause mass effects on adjacent structures. Common symptoms include blurred vision, diplopia, and headache, while intracerebral hemorrhage is less frequent than in intracerebral cavernous hemangiomas.^[1,3]^ Complete surgical removal is challenging due to their high vascularity.^[1,6]^

The differential diagnosis of cavernous sinus region tumors is wide^[7]^ (Table 1), with meningioma making up to 41% of masses in the cavernous sinus region; however, they often affect older adults, particularly women, with a peak occurrence in their 5th to 7th decade of life.^[1]^ Typically, meningiomas show homogeneous intense enhancement on gadolinium-enhanced MR images. These may also show restricted diffusion at MRI and appear hyperdense at CT scan owing to their cellular nature. Other features that may give a clue to this diagnosis include: calcification, vascularity, cystic areas, and hyperostosis of adjacent bone. Luminal narrowing of the ICA can be seen as well.^[1,8]^

**Table 1:** Summary of the characteristics of common cavernous sinus region tumors.

Parameter	Cavernous sinus hemangioma	Meningioma	Pituitary Adenoma	Schwannoma
Prevalence	Rare. Mostly affect middle-aged women	F > M, 50–70 yr	Adults. prolactinoma and corticotroph adenoma: F > M, GH–secreting adenoma: M > F	Sporadic (more common): 50–60 yr, Neurofibromatosis type 2: earlier age
Key distinguishing imaging features	Very bright on T2, strong progressive “filling-in” enhancement, no dural tail	Isointense, homogeneous intense enhancement, dural tail, hyperostosis common	Arises from sella, heterogeneous enhancement, sellar expansion, possible hemorrhage (apoplexy)	Along cranial nerve, often cystic/heterogeneous, foraminal widening, no dural tail
MRI intensity	T2 Markedly Hyperintense	T1 & T2 Iso/Hypointense to gray matter	T1 Hyperintense (if hemorrhagic)	T1 Hypointense & T2 Hyperintense
CT density	–	Hyperdense	–	–
Bone hyperostosis	–	+++	–	–
Enhancement intensity	*++++* (Homogenous/Heterogenous)	*++++* (Homogenous)	+ (Heterogeneous)	+ (Heterogeneous)
Constriction of the ICA	-	+++	–	–

CT = computed tomography, F = female, ICA = internal carotid artery, M = male, MRI = magnetic resonance imaging.

Schwannomas in the cavernous sinus are benign tumors arising from schwann cells of regional cranial nerves, mainly trigeminal nerve. They are more common in adults in their 5th and 6th decades of life and can be associated with neurofibromatosis type 2. At MRI these appear heterogeneously hyperintense on T2-weighted images and hypointense on T1 weighted images. These demonstrate heterogeneous enhancement as well. Clues to this diagnosis include: cystic areas within the tumor, fluid-fluid levels, and hemorrhage.^[1]^

CSHs are rare and occur more commonly in middle-aged women. Preoperatively, distinguishing CSH from meningioma or schwannoma depends on specific imaging features. While CSH lesions show high signals on T2-weighted and FLAIR MR images, the pivotal diagnostic clue was the enhancement pattern on dynamic contrast-enhanced T1-weighted MR images, which showed a characteristic progressive “fill-in” of contrast. This pattern, which leads to intense homogeneous enhancement on late gadolinium-enhanced MR images, differs from the typically uniform enhancement of meningiomas and the heterogeneous enhancement of schwannomas. Furthermore, another key imaging feature that distinguished CSH from meningioma was that CSH encases the ICA without causing the luminal narrowing typically associated with meningiomas.1 Other imaging modalities can be of benefit in the diagnosis of CSH, Scintigraphic imaging (99mTc pertechnetate–labeled red blood cells) can demonstrate accumulation of tracer within the lesion.^[1]^

These specific imaging features were crucial in suspecting CSH preoperatively. Three subtypes of extracerebral cavernous hemangiomas have been described based on gross, histopathology characteristics with variable MRI appearance, Type A – Encapsulated, soft, and compressible, composed of thin-walled vascular sinusoids. Type B – Solid and granular, with intervening vasculature and connective tissue. Type C – A combination of both types. Type A showed homogenous enhancement on MRI while types B and C showed markedly heterogeneous enhancement.^[5]^ Our patient’s tumor exhibited characteristics consistent with Type A cavernous hemangioma according to above-mentioned classification.^[5]^

In terms of presentation, our case aligns with previously reported cavernous hemangiomas of the sellar region, particularly in the presence of progressive visual decline and headaches. Most cases in the literature describe visual disturbances, including blurred vision, diplopia, and optic nerve compression, as common presenting symptoms.^[2,3]^ While oculomotor nerve palsy has been reported in large lesions,^[9]^ its presence in our case suggests significant cavernous sinus involvement. Notably, seizures and altered consciousness, which may be seen in other intracranial vascular malformations like meningiomas due to cortical irritation,^[10]^ were absent in this case.

Imaging findings in our case were consistent with previous reports, demonstrating a well-circumscribed, homogeneously enhancing sellar mass.^[1]^ Encroachment on the cavernous sinus and ICA is commonly described in similar cases.^[1]^ While most lesions exhibit homogeneous enhancement on MRI, some cases show heterogeneous enhancement, particularly in specific pathological subtypes.^[5]^

Regarding surgical management, the highly vascular nature of the tumor posed significant intraoperative challenges, necessitating a shift from the initial endoscopic transnasal transsphenoidal approach to a left pterional craniotomy. This aligns with literature suggesting that while a transsphenoidal approach may be attempted, it is often inadequate for large or parasellar-extending lesions. Open craniotomy is generally preferred for extensive or cavernous sinus-invading lesions.^[11]^

At the same time, intracranial CSH, anatomical constraints affect the morbidity rate, which reaches 38% due to its proximate relation to critical neurovascular structures; and recent studies point a decrease in this rate to 12.5% with modern microsurgical interventions, and subtotal resection is preferred to avoid excessive risk and preserve function^[9]^

Consequently, complete resection is often not feasible, with most cases favoring a subtotal resection or biopsy-based approach to minimize intraoperative hemorrhage and neurological complications.^[5]^

The intraoperative shift from an endoscopic transnasal approach to a pterional craniotomy due to unexpected vascularity and CSF leak directly illustrates the surgical challenges reported in the literature for large CSH extending into the parasellar space. While endoscopic approaches are feasible for smaller, purely sellar lesions, our experience aligns with reports favoring open craniotomy for managing significant cavernous sinus involvement and minimizing catastrophic hemorrhage.^[11]^

Histopathological analysis confirmed a benign vascular lesion characteristic of cavernous hemangiomas, composed of large, dilated vascular spaces without atypia or prior hemorrhage. This aligns with descriptions in the literature, which classify these lesions as vascular malformations rather than true neoplasms.^[1]^ A hallmark feature is the presence of thin-walled, dilated vascular spaces lacking smooth muscle or elastic lamina. Notably, unlike intracerebral cavernous malformations, sellar cavernous hemangiomas rarely contain hemosiderin deposits or evidence of prior hemorrhage, likely due to their distinct vascular composition.^[12]^

Postoperatively, our patient exhibited persistent ophthalmoplegia with gradual improvement, which is a commonly reported outcome.^[1]^ Literature suggests that postoperative cranial nerve deficits, particularly involving the oculomotor and abducens nerves, are frequently observed.^[1]^ However, the absence of postoperative CSF leaks or hemorrhage in our case is notable.

Despite this, vigilant postoperative monitoring for potential complications such as hydrocephalus is crucial following intraoperative CSF leakage, as it is a known neurosurgical risk.^[13]^ Overall neurological function remained stable, supporting reports that subtotal resection combined with conservative management generally leads to favorable outcomes.^[9]^

In terms of long-term prognosis, our patient demonstrated significant neurological improvement, reinforcing findings that subtotal resection is typically associated with positive long-term outcomes.^[9]^ While some cases require adjunctive therapy, such as radiotherapy, for residual lesion control^[9,12]^ our patient remains under surveillance with no immediate need for further intervention.

While factors predicting surgical outcomes and neurological recovery, such as preoperative neurological status, are increasingly well-defined for cavernous malformations of the brainstem^[14]^ and spinal cord; in a case series, patients with spinal CSHs underwent total resection and had significant neurological recovery postoperatively, and a long-term follow up of 55 months showed no recurrences, no major morbidities or mortalities, indicating that complete resection is possible.^[15]^

Similar prognostic data specifically for CSHs remain limited due to the rarity of these lesions. However, our case’s positive overall result and good neurological and visual recovery also suggest that the degree of preoperative deficits has a significant impact on prognosis.

This report, as a single case study, inherently limits the generalizability of its findings. While it offers valuable insights into the diagnosis and management of a rare condition, the observations may not be universally applicable. Furthermore, the relatively short follow-up period restricts our ability to assess long-term outcomes, including potential recurrence or complete neurological recovery. Diagnostic and treatment pathways can also be influenced by regional healthcare access and disparities, a common issue in underserved areas.^[4]^ Moreover, global health crises can further affect neurological care access, as evidenced by disruptions during the COVID-19 pandemic in various regions.^[16]^ Due to practical considerations, a direct patient perspective detailing her experience with the diagnosis, treatment, and recovery could not be formally included in this report. However, the patient has consistently expressed satisfaction with her overall recovery trajectory and remains compliant with follow-up appointments.

This case adds to the limited number of reported parasellar cavernous hemangiomas and highlights key diagnostic and surgical challenges associated with these tumors. emphasizing the importance of recognizing imaging characteristics to distinguish these lesions from meningiomas or pituitary adenomas,^[1]^ preventing misdiagnosis, while maintaining a tailored and less extensive approach in dealing with large lesions intraoperatively with high flexibility,^[17]^ especially in underserved health settings with limited advanced resources, where diagnostic challenges emerge, and the reliance on basic imaging techniques increases, while the access for advanced surgical interventions like embolization is limited, therefore, this case offers a relevant insight for neurosurgeons and radiologists who may encounter such challenges, and increases their recognition to consider CSHs in the differential diagnoses of sellar and parasellar masses besides other more common etiologies like meningiomas, which helps prevent catastrophic surgical consequences.

Additionally, intraoperative adaptability must be taken into account because unexpected vascularity may require conversion from transsphenoidal to open craniotomy. Moreover, keep in mind that subtotal resection can minimize neurological deficits while maintaining tumor control,^[9]^ and long-term surveillance is essential to monitor for any residual tumor growth, which may necessitate adjuvant therapy like radiotherapy in some cases.^[9,12]^

## 4. Conclusion

CSH, though benign, poses significant diagnostic and surgical challenges due to its rarity, high vascularity, and ability to mimic other sellar and parasellar masses. Its highly vascular nature necessitates a high index of suspicion when encountering sellar and parasellar tumors, influencing treatment approaches. Surgical resection remains the main treatment choice using different approaches. Better knowledge of CSH including its features and treatment options along with an integrated approach, including surgical intervention, preoperative imaging interpretation, multidisciplinary planning, and intraoperative flexibility and appropriate postoperative care, can ensure the patient’s optimal health and improvement.

## Acknowledgments

We would like to extend our thanks to everyone involved in gathering data and helping to validate this case report.

## Author contributions

**Conceptualization:** Omar H. Salloum.

**Resources:** Mohanad A. Abuzahra, Malak Hroub.

**Visualization:** Maha Ramzi.

**Writing – original draft:** Mohammed A. Barakat, Omar Hamadi, Maha Ramzi.

**Writing – review & editing:** Mohanad A. Abuzahra, Malak Hroub, Omar H. Salloum.

## References

[1] Mahalingam HV Mani SE Patel B. Imaging spectrum of cavernous sinus lesions with histopathologic correlation. Radiographics. 2019;39:795–819. 30978149 10.1148/rg.2019180122

[2] Hassanzadeh S Gao L Alvarado AM Camarata PJ Lakis NS Haeri M. Extra-axial cavernous angioma: a case report and review of the literature. Neurol Int. 2024;16:162–85. 38251058 10.3390/neurolint16010010 PMC10801606

[3] Sahin MC Bozkurt OF Sahin MM Celtikci E. Cavernous sinus capillary hemangioma: case report and literature review. Brain Spine. 2023;3:101776. 38021022 10.1016/j.bas.2023.101776 PMC10668058

[4] Uwishema O Boon P. Bridging the gaps: addressing inequities in neurological care for underserved populations. Eur J Neurol. 2025;32:e70073. 39912252 10.1111/ene.70073 PMC11799841

[5] Yao Z Feng X Chen X Zee C. Magnetic resonance imaging characteristics with pathological correlation of cavernous malformation in cavernous sinus. J Comput Assist Tomogr. 2006;30:975–9. 17082705 10.1097/01.rct.0000221953.06135.3e

[6] Bakan AA Alkan A Kurtcan S. Cavernous sinus: a comprehensive review of its anatomy, pathologic conditions, and imaging features. Clin Neuroradiol. 2015;25:109–25. 10.1007/s00062-014-0360-0 25410584

[7] Raheja A Couldwell WT. Cavernous sinus meningioma with orbital involvement: algorithmic decision-making and treatment strategy. J Neurol Surg B Skull Base. 2020;81:348–56. 33072476 10.1055/s-0040-1715471 PMC7561463

[8] Battal B Zamora C. Imaging of skull base tumors. Tomography. 2023;9:1196–235. 37489465 10.3390/tomography9040097 PMC10366931

[9] Noblett D Chang J Toussi A Dublin A Shahlaie K. Hemangioma of the cavernous sinus: a case series. J Neurol Surg Rep. 2018;79:e26–30. 29707473 10.1055/s-0038-1641731 PMC5919774

[10] Shariff S Nouh HA Inshutiyimana S. Advances in understanding the pathogenesis of epilepsy: unraveling the molecular mechanisms. Health Sci Rep. 2024;7:e1896. 38361811 10.1002/hsr2.1896 PMC10867297

[11] Nguyen DA Nguyen HT Duong TV Pham BH Vo HL. Surgical treatment of cavernous sinus cavernomas: evidence from Vietnam. Reports. 2020;3:16.

[12] Kuroedov D Cunha B Pamplona J Castillo M Ramalho J. Cerebral cavernous malformations: typical and atypical imaging characteristics. J Neuroimaging. 2023;33:202–17. 36456168 10.1111/jon.13072

[13] Sanker V Kundu M El Kassem S. Posttraumatic hydrocephalus: recent advances and new therapeutic strategies. Health Sci Rep. 2023;6:e1713. 38028696 10.1002/hsr2.1713 PMC10652704

[14] Chotai S Qi S Xu S. Prediction of outcomes for brainstem cavernous malformation. Clin Neurol Neurosurg. 2013;115:2117–23. 23962756 10.1016/j.clineuro.2013.07.033

[15] Zhao L Jiang Y Wang Y Bai Y Sun Y Li Y. Spinal epidural cavernous hemangiomas: a clinical series of 9 cases and literature review. Front Oncol. 2021;11:572313. 33816222 10.3389/fonc.2021.572313 PMC8010302

[16] Uwishema O Frederiksen KS Correia IFS Mahmoud A Onyeaka H Dost B. The impact of COVID‐19 on patients with neurological disorders and their access to healthcare in Africa: a review of the literature. Brain Behav. 2022;12:e2742. 35951730 10.1002/brb3.2742 PMC9480907

[17] Yilmaz H Akcay E Tabanli A. Is unilateral extended pterional craniotomy adequate instead of bicoronal (bifrontal) craniotomy in large or giant olfactory groove meningiomas? Turk Neurosurg. 2025;35:56–61. 39651883 10.5137/1019-5149.JTN.46246-24.3

